# Cancer-Related Malnutrition and Oxidative Stress in Colorectal Cancer Surgery: A Narrative Review of Pathophysiology and Postoperative Outcomes

**DOI:** 10.3390/antiox14111289

**Published:** 2025-10-28

**Authors:** Andrii Zahorodnii, Alicja Jelska, Paulina Głuszyńska, Hady Razak Hady

**Affiliations:** 1 Doctoral School, Medical University of Bialystok, 15-089 Bialystok, Poland; 2 2nd Clinical Department of General, Gastroenterological and Oncological Surgery, Medical University of Bialystok, 15-089 Bialystok, Poland

**Keywords:** colorectal cancer surgery, cancer-related malnutrition, oxidative stress, postoperative complications, perioperative nutritional support

## Abstract

Colorectal cancer (CRC) is a major global health concern with high morbidity and mortality, particularly complicated by postoperative complications. This narrative review explores the interplay between cancer-related malnutrition (CRM) and oxidative stress (OS) as key modifiable risk factors influencing surgical outcomes in CRC patients. Drawing on the recent literature, the article analyzes the multifactorial pathophysiology of CRM, including systemic inflammation, micronutrient deficiency, and metabolic derangements, and its role in weakening antioxidant defenses. Concurrently, oxidative stress, marked by elevated reactive oxygen species and impaired redox homeostasis, is linked to impaired wound healing, infection susceptibility, and anastomotic complications. The review highlights that CRM and OS are interdependent, synergistically exacerbating postoperative morbidity. It also emphasizes the diagnostic and therapeutic implications of integrating nutritional screening tools with oxidative biomarkers to optimize perioperative care. Current evidence suggests that early immunonutrition- and redox-modulating strategies may improve surgical resilience, though standardization of biomarkers and intervention protocols remains a challenge. This article underscores the need for individualized, integrated approaches to perioperative management and proposes CRM–OS interaction as a potential predictive model for surgical risk in CRC. The findings advocate for future clinical trials targeting both nutrition and oxidative status to enhance recovery and long-term prognosis.

## 1. Introduction

Colorectal cancer (CRC) is the third most diagnosed malignancy and the second leading cause of cancer-related deaths worldwide, posing a significant public health challenge [1,2,3,4]. In recent decades, the global incidence of CRC has increased, particularly in younger populations, probably due to dietary patterns, sedentary lifestyles, and microbiota alterations [5,6,7]. Surgical interventions remain the cornerstone of treatment in localized and locally advanced CRC, with curative intent often requiring multimodal strategies including chemotherapy and/or radiotherapy [1,8].

Despite improvements in perioperative care and the increasing adoption of enhanced recovery after surgery (ERAS) protocols, postoperative complications remain a frequent and severe problem [9,10]. Complications such as surgical site infections, anastomotic leakage, delayed wound healing, and prolonged hospitalization not only impair recovery but may also negatively impact long-term oncologic outcomes [11]. Consequently, the recent literature increasingly highlights the importance of analyzing modifiable risk factors, with particular focus on nutritional status and oxidative stress [12].

Cancer-related malnutrition (CRM), affecting a significant number of gastrointestinal cancer patients, is a clinically relevant but frequently underrecognized condition [13]. It has been shown to influence immune function, response to treatment, and wound healing capacity. One of the key biological processes associated with the adverse effects of CRM is oxidative stress (OS)—a metabolic disturbance characterized by an excess of reactive oxygen species (ROS) and/or insufficient antioxidant defense [14]. In the surgical setting, OS has been linked to endothelial dysfunction, impaired tissue regeneration, and increased susceptibility to postoperative infections and anastomotic complications [15].

Understanding the interplay between malnutrition, oxidative stress, and surgical outcomes may help identify high-risk patients, improve perioperative care, and optimize recovery. This review aims to synthesize current knowledge regarding the role of cancer-related malnutrition in modulating oxidative stress parameters and the implications for postoperative complications in patients undergoing surgery for colorectal cancer.

### Literature Search Approach

This work represents a narrative review based on a targeted search of recent studies. The literature search was performed between January and October 2025 using PubMed, Scopus, NCIM/NLM databases, and Google Scholar, with additional consultation of global epidemiology sources (e.g., GLOBOCAN/WHO fact sheets and updates). The search combined key terms related to colorectal cancer, malnutrition, and oxidative stress, including: “colorectal cancer,” “malnutrition,” “GLIM,” “oxidative stress,” “redox balance,” “reactive oxygen species,” “immunonutrition,” and “surgery/ERAS”. References cited in relevant review papers and clinical guidelines were also screened manually to identify additional sources.

To ensure recency and clinical relevance, we prioritized publications from the last five years (2020–2025), incorporating older seminal papers essential for mechanistic or methodological context. Inclusion criteria covered studies in adult colorectal cancer populations that reported nutritional status or interventions and/or oxidative stress parameters in relation to perioperative/postoperative outcomes. We excluded non-CRC populations without extractable CRC data, animal studies, non-English articles, and conference abstracts without full text.

In total, 88 publications were included in the qualitative synthesis, comprising original clinical studies, systematic reviews/meta-analyses, and guidelines/position papers directly addressing the interplay between cancer-related malnutrition, oxidative stress, and surgical outcomes in colorectal cancer. Given the heterogeneity of biomarkers, sampling time points, and interventions, the evidence was summarized descriptively, emphasizing convergent mechanistic links and clinical implications rather than pooled quantitative estimates.

## 2. Malnutrition in Colorectal Cancer—Clinical Relevance

CRM is highly prevalent among patients with gastrointestinal cancers; some studies report rates approaching 90%, with over 60% of patients requiring urgent nutritional intervention [16,17,18]. The pathogenesis of CRM is multifactorial, involving reduced oral intake (due to tumor location or treatment side effects such as nausea, dysgeusia, or mucositis), systemic inflammation, and catabolic metabolic alterations that lead to skeletal muscle breakdown and weight loss [19].

In colorectal cancer (CRC), malnutrition is often present at the same time of diagnosis, and its severity frequently worsens during treatment. All risk factors that contribute to nutritional decline include tumor-induced anorexia, gastrointestinal obstruction, chemotherapy-induced gastrointestinal toxicity, and psychological distress. Notably, CRM is not limited by the severity of the disease. Patients with potentially curable tumors may also be at significant nutritional risk due to aggressive multimodal therapies [20].

From a surgical standpoint, malnourished patients are consistently shown to have a higher risk of postoperative complications [21]. Protein-energy deficiency compromises collagen synthesis, impairs angiogenesis, and delays tissue remodeling, leading to delayed wound healing, an increased risk of infection, and anastomotic dehiscence [22,23,24]. Furthermore, malnutrition reduces the patient’s tolerance to oncological treatments and may negatively affect immune response, thereby impairing long-term prognosis [25].

Early identification of malnutrition is essential, and it has been validated by screening tools, such as the Nutritional Risk Screening (NRS 2002), Subjective Global Assessment (SGA), and the GLIM criteria. However, clinicians often fail to appreciate the significance of CRM, leading to nutritional support being initiated later in the treatment course than is optimal [26]. 

Preoperative nutritional intervention has been associated with improvement of the surgical outcomes and reduced complication rates in high-risk patients [27]. Nutritional strategies, including enteral or parenteral support, correction of micronutrient deficiencies, and immune-modulating supplementation (e.g., arginine, omega-3 fatty acids, nucleotides), may strengthen immune function and mitigate oxidative damage during the perioperative phase [28].

## 3. Oxidative Stress in Colorectal Cancer: Mechanisms and Clinical Impact

### 3.1. Definition and Molecular Basis

OS is defined as a disruption of redox homeostasis characterized by excessive production of reactive oxygen species (ROS) and/or compromised antioxidant defenses, resulting in cumulative molecular damage [29]. ROS are natural byproducts of mitochondrial metabolism, which play a role in normal physiological signaling. However, excessive ROS levels or insufficient antioxidant capacity can disrupt cellular redox homeostasis, promoting inflammatory and degenerative processes [30].

The antioxidant defense system consists of enzymatic components- such as superoxide dismutase (SOD), catalase (CAT), glutathione peroxidase (GPx), and non-enzymatic molecules of both endogenous (e.g., glutathione (GSH), uric acid, melatonin) and dietary origin (e.g., vitamins C and E, carotenoids, polyphenols) [31,32]. These systems work synergistically to neutralize ROS and protect cellular structures. A sustained redox imbalance contributes to oxidative injury, mitochondrial dysfunction, and systemic inflammation. It has been implicated in the pathogenesis of numerous inflammatory diseases, including cancer [33].

### 3.2. Oxidative Stress in Gastrointestinal Cancers

OS plays a key role in the development and progression of gastrointestinal cancers, including CRC [3,34]. Elevated levels of ROS can promote carcinogenesis by inducing DNA damage, activating proto-oncogenes, inhibiting tumor suppressor genes, as well as enhancing cancer cell proliferation and resistance to apoptosis [3,8,35]. In CRC, the Nrf2–KEAP1 pathway plays a dual role: while its proper activation supports antioxidant defense via antioxidant response elements (ARE), persistent or dysregulated Nrf2 signaling may promote tumor cell survival, therapy resistance, and progression. In parallel, constitutive NF-κB activation drives inflammation-mediated tumor development [36]. Inflammation-driven ROS production further contributes to the tumor microenvironment, facilitating angiogenesis and metastasis [2].

Cancer cells often exhibit an internal redox imbalance due to mitochondrial dysfunction and increased metabolic demands, which leads to both local (tumor-related) and systemic oxidative stress. In CRC, elevated oxidative stress markers, such as malondialdehyde (MDA) and 8-hydroxy-2′-deoxyguanosine (8-OHdG), and decreased antioxidant levels have been correlated with tumor aggressiveness, advanced disease stage, and a worse prognosis [37,38,39,40,41].

Moreover, anticancer therapies such as chemotherapy and radiotherapy can further amplify oxidative stress, contributing to tissue toxicity and systemic side effects [3,8]. Although ROS generation is part of the intended cytotoxic mechanism, excessive or uncontrolled redox stress may damage healthy tissues and exacerbate cancer-related complications [42].

### 3.3. Oxidative Stress and Surgical Outcomes

Surgical procedures induce a complex systemic response characterized by inflammation, ischemia–reperfusion injury, as well as activation of immune and neuroendocrine pathways. These processes lead to the overproduction of ROS, especially at the site of tissue damage, and contribute to a temporary but significant redox imbalance [43,44,45]. The intensity of oxidative stress has been associated with the extent of surgical trauma and has emerged as a potential predictor of postoperative complications.

Oxidative stress impairs wound healing by disrupting the function of fibroblasts, inhibiting collagen synthesis, and impairing angiogenesis. ROS excess can also damage endothelial cells, promote local inflammation, and increase the risk of anastomotic leakage or surgical site infections [46,47]. In CRC surgery, these effects are of considerable clinical significance owing to the anatomical complexity and the inherent risk of contamination associated with colorectal procedures [47].

Elevated levels of oxidative stress markers such as MDA and 8-OHdG, accompanied by reduced concentrations of GSH and total antioxidant capacity (TAC), have been associated with impaired wound healing and increased susceptibility to postoperative complications [3,47,48]. Based on these observations, it is reasonable to hypothesize that persistent redox imbalance may also contribute to prolonged hospital stays and less favorable postoperative recovery. Monitoring perioperative redox status may help identify patients at higher risk and guide nutritional or pharmacologic interventions aimed at modulating oxidative damage.

The magnitude of oxidative stress after colorectal cancer surgery also depends on the surgical approach. Minimally invasive techniques, such as laparoscopic and robotic-assisted surgery, are associated with reduced tissue trauma, milder inflammatory response, and lower postoperative oxidative burden compared with conventional open procedures. Several recent studies have demonstrated that levels of oxidative biomarkers, including MDA and 8-OHdG, rise less markedly after laparoscopic resection, while TAC remains better preserved [49,50,51]. This redox advantage may partially explain the faster recovery, shorter hospital stay, and lower complication rates typically observed in minimally invasive colorectal surgery.

### 3.4. Biomarkers of Oxidative Stress in Colorectal Cancer Patients

In clinical practice, oxidative stress is typically evaluated using indirect biochemical markers that indicate the relationship between prooxidant activity and antioxidant defenses. In CRC patients, particularly those undergoing surgery, several markers have been investigated as potential indicators of tissue damage, redox imbalance, and clinical prognosis [52] (Table 1).

Some studies have introduced composite indices, such as the oxidative stress index (OSI)—calculated as the ratio between total oxidant status (TOS) and total antioxidative capacity (TAC) [58].

pite variability in methodology and standardization, perioperative monitoring of oxidative biomarkers may help identify patients at increased risk of complications, particularly those with pre-existing cancer-related malnutrition, where redox imbalance is often pronounced.

## 4. Pathophysiological Interactions Between Cancer-Related Malnutrition and Oxidative Stress

### 4.1. Overview of the Relationship Between CRM and OS

CRM and oxidative stress are highly prevalent and interdependent phenomena in patients with CRC, particularly in the perioperative setting. While CRM primarily reflects a deficiency in macro- and micronutrients, OS results from an imbalance between prooxidant activity and antioxidant defenses. Both conditions are independently associated with impaired immune response, reduced therapy tolerance, and delayed postoperative recovery.

Emerging evidence suggests that these two processes are pathophysiologically linked. CRM contributes to oxidative imbalance through nutrient deficiencies, particularly in proteins, selenium, zinc, and antioxidant vitamins, which impair the endogenous redox system [34,59,60,61]. Persistent oxidative stress may promote metabolic derangements, systemic inflammation, and tissue catabolism, thereby potentiating malnutrition [62].

This bidirectional relationship is particularly relevant in surgical patients, where adequate nutritional and redox status are essential for wound healing, immune surveillance, and overall surgical resilience. Understanding this interaction provides a rationale for integrating nutritional and redox-targeted strategies into perioperative care.

### 4.2. Deficient Antioxidant Defenses in CRM

One of the key mechanisms linking cancer-related malnutrition (CRM) with oxidative stress (OS) is the deficiency of substrates essential for proper antioxidant defense, as described in Section 3.1. In malnourished patients, reduced intake or absorption of critical micronutrients such as selenium, zinc, and antioxidant vitamins (A, C, and E) compromises redox homeostasis and increases susceptibility to oxidative damage.

In addition to enzyme inactivation, CRM is associated with a marked reduction in non-enzymatic antioxidants, notably reduced glutathione. GSH is a key intracellular molecule that acts as a redox buffer, directly neutralizing ROS. Low levels of GSH have been observed in both plasma and tumor tissues of malnourished cancer patients, suggesting systemic oxidative vulnerability [63].

This compromised antioxidant capacity may contribute to elevated oxidative damage and inefficient wound healing in the perioperative setting. Importantly, patients with pre-existing nutritional deficiencies often demonstrate blunted redox responses to surgical stress, which may partially explain the higher incidence of complications in this group [64].

### 4.3. Metabolic Derangements and Oxidative Load

CRM is characterized by profound alterations in energy metabolism, including increased resting energy expenditure, enhanced proteolysis, and lipolysis. These catabolic changes not only drive weight loss and muscle wasting but also contribute to excessive generation of oxidative intermediates [65].

Protein degradation results in the release of iron and other prooxidant metals, which catalyze ROS formation via Fenton-type reactions (a process in which ferrous iron ions Fe^2+^ react with hydrogen peroxide H_2_O_2_, producing hydroxyl radicals •OH) [66]. Concurrently, lipid mobilization increases the availability of polyunsaturated fatty acids, which are prone to peroxidation, leading to the accumulation of cytotoxic byproducts, such as MDA and 4-HNE. These compounds exacerbate oxidative tissue damage and perpetuate cellular stress responses.

Moreover, glucose metabolism in malnourished cancer patients is often altered, with an increased reliance on anaerobic glycolysis (the Warburg effect), which can affect the balance of NADPH and redox buffering systems. Together, these metabolic disturbances amplify oxidative load and weaken the host’s ability to counteract redox imbalance, especially during the physiological demands of surgery.

### 4.4. Perioperative Clinical Implications

**Figure 1:**
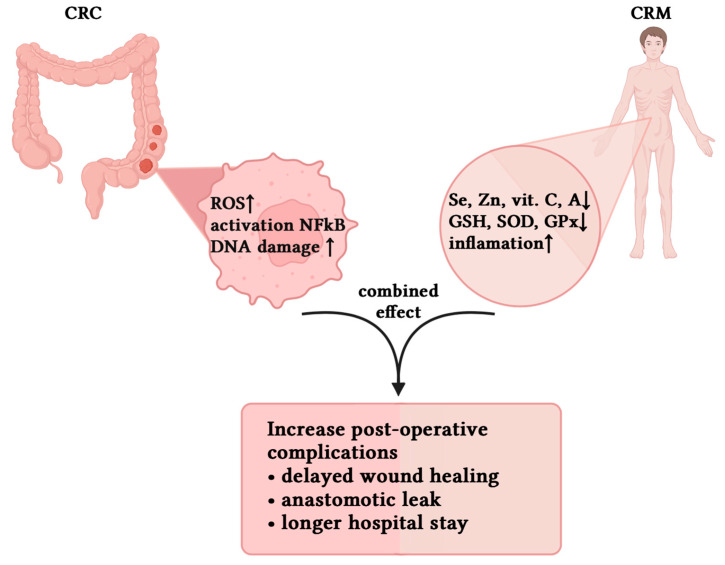
Interplay between tumor-related oxidative stress and systemic antioxidant deficiency leading to increased postoperative complication rates in CRC patients. Black arrows indicate the causal flow: tumor-related oxidative stress and CRM-related systemic antioxidant deficiency converge (curved arrows) and lead to increased postoperative complications (downward arrow).

The interplay between CRM and OS represents a clinically relevant risk factor in the perioperative management of CRC patients (Figure 1). Both conditions are independently associated with delayed wound healing, reduced immune function, and increased rates of complications. When occurring together, as they frequently do, they may exert synergistic adverse effects that compromise surgical outcomes.

Despite this, CRM is often underdiagnosed, and OS is rarely monitored in routine clinical care [67,68]. This diagnostic gap may result in missed opportunities for risk stratification and timely intervention. Integrating nutritional assessment with biochemical markers of redox balance could help identify patients at elevated risk for postoperative morbidity.

Moreover, if the causal relationship between CRM and OS is confirmed in future clinical studies, it may justify the development of targeted perioperative interventions, such as antioxidant supplementation, immunonutrition, or redox-modulating therapies, to mitigate oxidative injury and improve recovery. This would represent a paradigm shift in the preoperative optimization of high-risk patients undergoing surgical oncology procedures.

As oxidative stress and redox imbalance become increasingly investigated as therapeutic targets, the identification of patients with concurrent CRM and OS may help guide the design of future clinical trials. In addition to its biological relevance, this interaction could serve as a useful preoperative risk assessment tool, supporting individualized nutritional and pharmacological decision-making in perioperative care.

The growing body of clinical evidence supports the association between CRM and/or OS and adverse postoperative outcomes in colorectal cancer patients. Table 2 summarizes representative clinical studies that have examined the impact of CRM and OS parameters on surgical morbidity, mortality, and long-term prognosis.

Collectively, these findings highlight the prognostic value of combined nutritional and redox assessment in the perioperative setting.

## 5. Perioperative Interventions in Colorectal Cancer: Nutrition and Redox Modulation

### 5.1. Nutritional Interventions: Evidence and Recommendations

Optimizing nutritional status is a cornerstone of perioperative management in CRC patients, particularly those at risk of malnutrition. Clinical guidelines recommend early screening using validated tools (e.g., NRS 2002, GLIM) and the initiation of individualized nutritional support, including oral nutritional supplements (ONS), enteral nutrition (EN), or parenteral nutrition (PN), depending on the patient’s functional status and gastrointestinal integrity [10,69,70,71].

In addition to standard support, the concept of immunonutrition has gained considerable attention [28,72,73]. This approach involves supplementing the diet with specific nutrients such as arginine, omega-3 fatty acids, and nucleotides, which modulate immune responses, reduce inflammation, and support antioxidant defenses. Randomized trials and meta-analyses suggest that immunonutrition may reduce hospital stays, decrease surgical site infections, and lower complication rates in patients undergoing CRC surgery [72,73,74,75,76].

Importantly, perioperative nutritional therapy should not be delayed until after surgery. Prehabilitation protocols now recommend initiating support 7–14 days before the operation, especially in patients with severe malnutrition or systemic inflammation. Timely nutritional intervention may not only improve surgical tolerance but also attenuate oxidative stress, creating a more favorable metabolic environment for recovery.

### 5.2. Modulating Oxidative Stress—Current Concepts and Challenges

While nutritional therapy remains the foundation of perioperative optimization in CRC patients, the role of targeted modulation of OS is receiving increasing attention. Several micronutrients with antioxidant properties, including vitamins C and E, selenium, zinc, and N-acetylcysteine, have been investigated for their potential to improve redox balance and support recovery after surgery [31,77]. However, clinical evidence remains heterogeneous, and routine antioxidant supplementation is not currently recommended in most guidelines [78,79,80,81,82].

One of the challenges in this field is the complexity of redox biology. ROS are not only harmful byproducts but also serve important signaling functions in immune defense and wound healing. Blunting oxidative responses too aggressively may paradoxically impair physiological recovery. Therefore, any redox-targeted therapy must be carefully dosed and timed, ideally tailored to the patient’s baseline redox status and clinical condition.

Emerging strategies include the use of redox-sensitive biomarkers to identify patients with excessive oxidative burden and guide antioxidant interventions. Trials of perioperative antioxidant-enriched nutrition, as well as pharmacological agents with redox-modulating effects, are ongoing. Although no consensus protocols exist to date, the concept of selectively attenuating pathological OS without disrupting physiological ROS activity represents a promising frontier in perioperative care.

#### The Antioxidant Paradox: Risks of Overcorrection

Although antioxidant therapies hold promise in mitigating excessive oxidative stress, recent research has underscored a paradox: under certain conditions, antioxidant supplementation can disrupt physiological redox signaling and even promote tumor progression. This so-called antioxidant paradox highlights the complex and context-dependent role of ROS in cancer biology and perioperative recovery [83].

Excessive suppression of ROS by high-dose antioxidant therapy—particularly in patients without marked oxidative imbalance—may therefore hinder these physiological responses, delay tissue repair or weaken immune defense [84,85].

Moreover, in oncology, ROS are involved in inducing apoptosis of malignant cells and enhancing the cytotoxic effects of chemotherapy and radiotherapy. Several experimental and clinical studies have demonstrated that indiscriminate antioxidant supplementation may reduce treatment efficacy by protecting tumor cells from ROS-mediated apoptosis [86]. For instance, inhibition of ROS signaling through vitamin E or N-acetylcysteine was shown to accelerate intestinal tumor growth in murine models. Similarly, persistent activation of the Nrf2 pathway—a major antioxidant regulator—has been associated with increased chemoresistance and poorer clinical outcomes in colorectal cancer [86].

Therefore, the use of antioxidants in oncologic and perioperative contexts requires a balanced and individualized approach. Instead of routine supplementation, redox-modulating strategies should be guided by biochemical assessment of oxidative stress markers and tailored to patient-specific redox profiles. This precision-based approach aims to correct pathological oxidative imbalance while preserving the physiological ROS signaling necessary for healing and host defense [34,87].

### 5.3. Combined Strategies and Individualized Perioperative Care

The multifactorial interplay between malnutrition and oxidative stress (OS) in surgical CRC patients supports the need for integrated, personalized perioperative strategies. Isolated interventions targeting nutrition or OS alone may not be sufficient to reverse the compounded biological risk. Instead, a combined approach that simultaneously addresses caloric and micronutrient deficits, inflammatory burden, and redox imbalance may offer better clinical outcomes.

Such strategies include preoperative screening using tools like SGA, NRS-2002, or GLIM, combined with targeted laboratory markers of redox status (e.g., MDA, GSH, TAC) to stratify risk. Nutritional prehabilitation, especially immunonutrition, and early postoperative support should be tailored to the patient’s nutritional and inflammatory profile [88]. For selected patients, antioxidant-enriched formulas or redox-active pharmacotherapy may further improve outcomes, although more robust clinical trials are needed to standardize these protocols.

Ultimately, integrating nutritional and redox considerations into the surgical care of CRC patients represents a shift toward more individualized perioperative medicine. Future pathways may include algorithm-driven models that combine nutritional risk scores with oxidative stress biomarkers to guide decision-making and resource allocation.

## 6. Summary and Future Directions

### 6.1. Key Conclusions

Malnutrition and OS are common and clinically significant problems in patients undergoing surgery for colorectal cancer. Evidence indicates that CRM is associated with oxidative imbalance, likely mediated by micronutrient deficiencies, chronic inflammation, and catabolic metabolism. In turn, OS exacerbates immune dysfunction, impairs tissue repair, and increases the risk of postoperative complications. These two processes often coexist and interact synergistically, resulting in poorer clinical outcomes. Despite this, both CRM and OS remain underrecognized in routine surgical care.

### 6.2. Clinical Implications

The co-occurrence of CRM and OS supports a shift toward integrated perioperative management strategies. Early nutritional screening and individualized interventions, including immunonutrition and antioxidant support, may reduce surgical morbidity and improve recovery. Redox status, although not yet standard in clinical protocols, may become a valuable tool for risk stratification and personalized therapeutic planning in surgical oncology.

### 6.3. Research Gaps and Future Perspectives

Further research is needed to define reliable biomarkers of oxidative stress, standardize measurement protocols, and validate redox-modulating interventions in high-risk surgical patients. Clinical trials combining nutritional and antioxidant therapies, tailored to individual redox profiles, could offer a new dimension of perioperative care. Ultimately, the integration of CRM and OS into surgical risk models may support the development of evidence-based, individualized protocols to improve outcomes in CRC surgery.

## 7. Limitations

This review highlights important interactions between cancer-related malnutrition, oxidative stress, and surgical outcomes in colorectal cancer. However, several limitations must be acknowledged.

Firstly, there is significant variability in the methodology of available studies. Differences in how OS is assessed—ranging from the choice of biomarkers to sample handling, timing of collection, and analytical techniques—limit comparability between results. This lack of standardization makes it difficult to define clinically relevant thresholds and integrate OS assessment into everyday surgical practice.

Secondly, although a biological connection between CRM and OS is highly plausible, the evidence supporting a causal relationship remains limited. Most studies are observational and do not allow for definitive conclusions regarding the direction of interaction. Well-designed, prospective studies are needed to verify whether improving nutritional status can directly modulate oxidative balance and translate into better clinical outcomes.

Thirdly, the current body of evidence is not always specific to CRC. Many studies include mixed populations of patients with different gastrointestinal malignancies, which limits the generalizability of the findings to colorectal surgery alone.

Lastly, OS is a highly sensitive marker influenced by multiple non-cancer-related variables. Chronic conditions such as diabetes, cardiovascular or inflammatory diseases, smoking, and obesity can all affect baseline oxidative status. This increases the number of exclusion criteria in clinical studies and complicates the identification of cancer-specific OS patterns. In practical terms, it limits the use of OS as a standalone indicator unless these confounding factors are carefully controlled [12].

## Figures and Tables

**Table 1:** Markers of oxidative damage.

Oxidative Damage		Antioxidative Capacity	
		Enzymatic Defenses	
Malondialdehyde (MDA)	Byproduct of lipid peroxidation. Associated with oxidative damage to cell membranes [38,53].	Superoxide Dismutase (SOD)	An antioxidant that catalyzes the dismutation of superoxide radicals into oxygen and hydrogen peroxide [31,54].
4-Hydroxynonenal (4-HNE)	Reactive lipid peroxidation product that forms protein and DNA adducts [53].	Catalase (CAT)	An enzyme that decomposes hydrogen peroxide into water and oxygen, preventing oxidative damage [51,54,55].
8-Hydroxy-2′-deoxyguanosine (8-OHdG):	A marker of ROS-induced DNA damage, detectable in plasma and tumor tissue, correlated with cancer progression and recurrence risk [53].	Glutathione Peroxidase (GPx)	A selenium-containing enzyme that reduces hydrogen peroxide and lipid hydroperoxides using glutathione [51,54].
Advanced Oxidation Protein Products (AOPP)	Product of protein oxidation, elevated in CRC patients and linked to disease severity [53,56].	**Non-enzymatic antioxidants**	
		Glutathione (GSH)	Antioxidant tripeptide that neutralizes free radicals and maintains intracellular redox balance [51,54].
		Vitamins C, E, and A	neutralize free radicals, protect cell membranes from lipid peroxidation, and support immune function and tissue repair. Key non-enzymatic antioxidants [51,57].

**Table 2:** Summary of clinical studies linking CRM and OS with postoperative outcomes in CRC.

Study (Author, Year)	Study Design/Population	CRM or OS Parameters Assessed	Key Findings
Riad et al., 2023 [18]	Prospective multicenter cohort study (*n* = 5709)	Clinical diagnosis of malnutrition	Malnutrition was associated with a twofold increase 30-day postoperative mortality and increased complications
Lee et al., 2021 [23]	Retrospective cohort, CRC patients from US database (*n* = 11357)	Diagnosed malnutrition	Malnourished patients had a higher risk of complications, prolonged stay, and mortality
Shen et al., 2023 [21]	Prospective cohort, elderly CRC patients (*n* = 385)	GLIM-defined malnutrition	Malnutrition significantly increased risk of postoperative complications and lowered long-term survival
Song et al., 2022 [24]	Prospective cohort, CRC patients (*n* = 918)	GLIM criteria	Malnourished patients had significantly worse short-term surgical outcomes
Martínez-Escribano et al., 2022 [17]	Case–control study in elderly CRC patients	Clinical nutritional status	Malnutrition increased risk of surgical complications and adverse discharge outcomes
Leimkühler et al., 2022 [15]	Observational study, gastrointestinal cancer incl. CRC (*n* = 81)	Serum free thiols (OS marker)	Low preoperative serum thiol levels, reflecting increased systemic oxidative stress, were predictive of postoperative complications and prolonged hospital stay
Sawai et al., 2022 [48]	Retrospective study, CRC patients (*n* = 163)	d-ROMs (Derivatives-reactive oxygen metabolites)	High oxidative stress levels were associated with worse prognosis in CRC, independent of tumor stage
Boakye et al., 2020 [41]	Prospective cohort, CRC patients (*n* > 3300)	d-ROMs and total thiol level	High oxidative stress levels were strongly associated with poorer prognosis
Kang et al., 2023 [37]	Retrospective cohort study CRC patients (*n* > 564)	8-OHdG levels in tumor tissue	Low tumor expression of 8-OHdG was significantly associated with poorer 5-year event-free and disease-specific survival

## Data Availability

No new data were created or analyzed in this study. Data sharing is not applicable to this article.
